# Behind male Saudi nursing students’ mental health facade: a husserlian phenomenological approach

**DOI:** 10.1186/s12912-021-00779-w

**Published:** 2021-12-10

**Authors:** Ejercito Mangawa Balay-odao, Nahed Alquwez, Abdulellah Al Thobaity, Khalaf Al Otaibi, Yousef Ali Abdulrahman Alsakran, Jonas Preposi Cruz

**Affiliations:** 1 Department of Nursing, College of Applied Medical Sciences, Shaqra University, Al Dawadmi, Saudi Arabia; 2 College of Applied Medical Sciences, Taif University, Taif, Saudi Arabia; 3 Level 7, Department of Nursing, College of Applied Medical Sciences, Shaqra University, Al Dawadmi, Saudi Arabia; 4 Department of Medicine, School of Medicine, Nazarbayev University, Nur-Sultan, Kazakhstan

**Keywords:** Male saudi, Mental health facade, Nursing student, Saudi Arabia

## Abstract

**Background:**

Every person has a persona (or mask) which is the façade that every person shows to the world. Thus, males use façade to reveal or conceal their true feelings and emotions. Also, the male uses mental health façade to protect themselves from prejudice and judgment. Thus, the study aimed to explore the experiences of male Saudi nursing students of mental health.

**Method:**

Husserl’s descriptive phenomenology was used as a guiding lens to explore. Eleven participants were involved in the study by using the referral sampling technique. An unstructured interview was performed to gather information from the participants. The seven steps of the descriptive Colaizzi process were followed to investigate and examine the obtained data. The credibility, dependability, confirmability, transferability, and reflexivity criteria were observed to ensure the rigor of the study.

**Results:**

The findings have two major themes. The first theme is the unadulterated smile that describes optimism in the family and mutual guarantee. The second theme is the orchestrated smile, which describes avoiding diverting burdens, social responsibility, protection of self, and reputation.

**Conclusions:**

The findings document that the mental health façade of male Saudi nursing students is associated with the expectation of family optimism, mutual guarantee, the expectation of society, and self-protection.

## Introduction

Public awareness of mental health increases, and the male perspective is vital to explore [1]. Exploring the male perspective on mental health helps identify the effective mental health intervention, issues and barriers, and their coping mechanisms. Bilsker et al. [2] noted that men’s mental health is “hidden in plain sight” because they were less likely to express their feelings and emotions for unclear reasons. Baker [3] added that men are reluctant to accept their mental health issues and have their way of expressing their feelings. Conducting this study is of great help in implementing an approach to mental health policy and practice to promote positive mental health and avoid psychological problems.

Mental health is the complete welfare of individuals to cope with stressful situations to function effectively and contribute productively to their community and themselves [4]. Mental health or behavior is affected by people’s attitudes, beliefs, and practices [5]. Furthermore, cultural practices influence the perceptions and attitudes of individuals [6]. However, people often associate mental health with mental illnesses, which creates mental health stigma that hinders individuals from freely discussing their mental health, especially among men, due to negative notions, discrimination, and typecasts [7]. This mental health stigma results in individuals being silent about their mental health due to fear of being labeled as people with mental illness or treated differently in society [8], especially for men [6]. This notion shows that people use façade to portray emotions and reactions that are not the same as their real feeling on the inside. According to Carl Jung [9], every person has a persona (or mask) which is the façade that every person shows to the world. This façade of the person is their way to conceal their true feelings and emotions. Thus, men use mental health façade to protect themselves from prejudice and judgment [8]. Also, mental health façade is used to prevent emotional exhaustion, especially in an environment or situation where a person feels pressured [10].

Furthermore, psychological expression is uncommon for men [11], and this is associated with the adherence of men to their masculine role, which prevents them from seeking mental health and expressing their feelings [2]. Men tend to control and strive to suppress their feelings and emotions, negatively affecting their mental health [12]. Wong et al. [11] revealed that masculine norms reduce the mental health-seeking behavior of men that negatively affects their mental health. Breland et al. [13] further showed that men are doubtful to share their emotions and feelings even if they show manifestations of mental problems. The social contract of males is one of the reasons why identifying their mental health status is difficult [2]. This idea accounts for the high prevalence of male mental health issues, with nearly 8,000,000 committing suicides, wherein males have a higher prevalence than females [14].

Saudi Arabia remains highly patriarchal in nature. Despite the many advances and social changes that have been witnessed in Saudi Arabia in recent years, traces of hegemonic masculinity ideology still exist in society [15]. Hegemonic masculinity “refers to a societal pattern in which stereotypically male traits are idealized as the masculine cultural ideal, explaining how and why men maintain dominant social roles over women and other groups considered to be feminine” [16]. In Saudi society, males’ dominance is demonstrated by having the responsibility to be their family’s guardian and ensuring that their family’s honor is free from scandals. They assume the huge responsibility of ensuring the safety and security of their family starting from a young age and throughout their life [15]. Because of this status, society places high expectations on male Saudis to create and maintain a positive image in every decision and action that they are involved in [17].

Moreover, the country’s social rules and religion do not permit the mixing of genders. This sex segregation extends to all areas of society, including in nursing education [18]. Nursing education in the country is one of the few remaining in the world that is segregated by sex. Nursing students belonging to the opposite sex do not have the opportunity to learn and socialize with each other. Those who oppose sex segregation in education argue that this system has negative impacts on the psychosocial development of the students, which could also affect their mental health [19]. Also, previous studies have shown gender differences in nursing students’ mental health and well-being in Saudi Arabia [20–22]. These factors may have implications on the mental health of Saudi male nursing students.

However, information about the incidence of mental illness is limited in Saudi Arabia [23], and previous studies have limited samples (1). Al-sughar and Ferwana [24] reported that out of 354 participants, 41% of male adolescents experience mental illness. With the reported cases of mental health problems for men, no study has been conducted to understand the perception of male Saudi nursing students about their mental health. A low magnitude of community awareness is also observed on the mental health issues of Saudi males [1]. Moreover, the mental health of adolescents has not been given much attention in Arab countries, such as Saudi Arabia [25]. Thus, identifying the male perspective on mental health is important to understand the perceived concepts, meanings, and experiences of male Saudi nursing students regarding mental health.

### Aim of the study

This Husserl phenomenological qualitative study aimed to explore the experiences of male Saudi nursing students of mental health.

## Methods

### Study design

The researcher undertook Husserl’s qualitative phenomenological research to explore the experiences of male Saudi nursing students of mental health. Bracketing, reflexivity, analyzing, interpreting, and intuiting Husserl’s descriptive phenomenology component was observed throughout the study [26].

### Researcher characteristics

Five of the researchers are instructors of the University in Saudi Arabia, and one is a level six nursing student. As an instructor, we teach nursing students in the classroom and supervise them in the clinical setting. They have observed that nursing students have different perspectives, behaviors, and attitudes which influence their mental health. This observation prompted them to conduct this study. Four of the researchers are male Saudi nationals, and two are Filipino. To prevent bias, the two Filipino researchers conducted interviews with the students. The nursing student researcher was responsible for recruiting and getting the written consent of those students who were interviewed.

### Sample strategy and sampling

The referral sampling technique was used to select the participants. First, the researchers posted posters regarding the study on the posting areas on the campus. The posters contain the information about the study (i.e., purpose of the study, intended participants, expected participation, the research process) and invitation for participation. An electronic poster containing the same information described above was forwarded to potential participants to maximize the recruitment. The contact information of the primary investigator was also included in the poster for any questions about the study and voluntary enrollment of participants. As written in the posters, students who decided to participate are asked to contact the primary investigator. Second, the researchers asked those who first participated for any referrals to increase the enrollment in the study. The researchers approached the referred potential participants, and similar information about the study was provided. They were then invited to participate in the study. The 11 male Saudi nursing students participated in the study. The saturation point of the study was achieved on the fifth participant, but the researchers added another participant to ensure that the saturation point was achieved. All the sir nursing students participated in the study voluntarily and signed informed consent to signify their understanding of the study and their voluntary participation. The participants were selected on the basis of these criteria; (1) The participant should be a male Saudi national student nurse, and (2) The participant must be willing to participate and commit himself to a series of interview sessions. The exclusion criteria are female nursing students, non-Saudi national nursing students, and those unwilling to contribute to the research.

The researchers used the codes “Nursing Student 1, 2, 3, 4, 5, 6, 7, 8, 9, 10, and 11” to protect the identity of the participants. Nursing Student 1, 7, 10 is a 20-year-old level 3 student. Nursing Student 2, 3, 4, and 5 are 20-year-old level 6 nursing students. Nursing Student 6 is a 23-year-old level 6 student. Nursing Student 8 is a 22-year-old level 4 student. Nursing Students 9 and 11 are 24-year-old level 6 students.

### Data collection

An unstructured interview questionnaire was used to conduct the data with a general question of “What are your perceptions about mental health?” Succeeding queries were based on the responses of the participants. The interview lasted from 40 to 60 min, with a follow-up session to validate the identified themes. Due to physical distancing and restrictions, the interviews were conducted through Zoom. The interview time and date were based on the preferences or availability of the participants. Before the commencement of the interview, consent to participate and consent for recording the interviews were taken. The recorded interviews were coded, transcribed, and analyzed.

### Data analysis

The seven steps of the descriptive Colaizzi process were followed to investigate and examine the obtained data. These seven processes are as follows: (1) gather the participants’ perspectives by understanding and reading the data several times, (2) comprehensively understand the meanings of the participant experiences and identify relevant statements associated with the study, (3) formulate meaning associated with the study from the extracted important sentences. The researchers reflexively “bracketed” their presumptions to confirm that the meanings are relevant and support participants’ experiences (4) conceptualize or cluster identified themes supported by participants’ experiences, (5) develop and classify the outline of the concepts and themes in Step 4, (6) formulate the fundamental structure of the experiences of the participants or the phenomenon examined by abridging themes and ideas, and (7) verify or validate the elemental structures of all the participants to make sure that their experiences are well captured. The researchers reanalyzed and modified the previous steps to support the feedback of the participants [26].

### Rigor/trustworthiness

The credibility, dependability, confirmability, transferability, and reflexivity criteria (16) were observed to ensure the rigor or trustworthiness of the study. The researcher’s strategies to guarantee credibility are persistent observation, triangulation, prolonged engagement, and self-evaluation. To achieve a thick and rich description or set of information for confirmability, the researcher described the data on the basis of the context of the participants on their behaviors and experiences, not simply describing their behaviors and experiences. This approach makes the data meaningful for a reader or outsider. The researcher also described the research process starting from the conceptualization up to the reporting of the findings. The recorded data from the Zoom interviews were extracted and kept in a file on the researcher’s computer with a password. The records of the study were kept during the research process. Two experts evaluated the data collected to confirm the accuracy of the analysis, themes, and coding. Coding systems were utilized in the analysis process to enhance dependability. To ensure the reflexivity, the researcher’s conceptual lens, and the overt and covert assumptions, biases and values were acknowledged.

### Ethical considerations

 This study was approved by the Shaqra University Research Ethics Committee (ERC-SU-20,200,015). The study was conducted after securing written informed consent from all of the participants. The researchers assured all participants that all data and information were strictly confidential, and neither codes nor words were used in the study that can associate the results with the participants. Also, all methods were carried out in accordance with relevant guidelines and regulations.

## Results

The individual wears a different mask (persona) in various events, groups, or society [9]. Thus, this study talks about the mental health façade of male Saudi nursing students. Every person wears a mask as they deal with every individual depending on the situation. The finding shows that every participant wears a mask (persona) as they deal with events, situations, or individuals in their environment. This mask of the participant is described as their mental health façade to show or hide their values, identity, and feelings. Mental health façade describes the factors or reasons behind the expression of emotions of male Saudi nursing student participants who described two main themes of their mental health façade: the unadulterated and orchestrated smiles of their psychological well-being.

### Unadulterated smile

Unadulterated smile is the first theme that describes the façade of male Saudi nursing students. An unadulterated smile describes or shows the authentic emotions or behaviors in their unprejudiced environment. These participants identified that the expression of emotion is achieved because of optimism in the family and mutual guarantee.

*“I open my problem to my family because they understand me, especially my brother. With their help, I can handle the day without any worries.”* Nursing Student 3.

The participants mentioned that they easily express their feelings and emotions because their family is optimistic. Optimism in the family helps in the development of their positive mental health. In addition, the optimistic attitude of the family about mental health helps them overcome stressful situations and promote comfort.

*“My family understands me and is willing to listen when I have a problem. When I share my problem with them, it helps me get along with myself, my sense of happiness, and my ability to face the demands of life and have safety and security.”* Nursing Student 1.

In maintaining mental health, male nursing students mentioned the importance of mutual guarantee. Mutual guarantee is the reciprocating emphatic and sympathetic attitudes of persons that help them overcome stressful situations. In dealing with stressful situations, the participants recognize the importance of mutual understanding of feelings and emotions with a person they trust and with whom they share a quality relationship. Trust is built in mutual guarantee, which is the basic foundation for showing real feelings and emotions. In this manner, they can help and support each other to deal with stressful situations.

*“Yes, relating to others is important to help me handle my stress and make healthy choices. Understanding others develops mutuality wherein problem sharing becomes good and beneficial.”* Nursing Student 4.

*“I restore my self-confidence first, and then communicate with those I trust, and then act on the advice.”* Nursing Student 10.

On the other hand, as the participants show their real emotions, they also hide as protection from the environment. This face is described as an orchestrated smile.

### Orchestrated smile

The second theme that described the mental health faced of the male Saudi nursing students is the orchestrated smile. Orchestrated smile is the façade of participants when they are suppressing or faking their emotions. This theme shows that the behaviors and emotions of the participants are not in sync with their real feelings. The participants fake or suppress their emotions or feelings to protect their identity and sense of belongingness. The participants orchestrate their smiles to manage their unwanted feelings, thoughts, and emotions. Such emotions help them preserve their ego and avoid prejudices from other individuals and society.

*“Sometimes, I am not interested in sharing my problem because solutions do not come from other people, and I am afraid that they will only judge me.”* Nursing Student 5.

Avoidance of diverting burden is one of the reasons why the participants avoid sharing unwanted thoughts, feelings, and emotions. They believe that everyone has their problem, and sharing concerns or unloading unwanted emotions can only cause a burden to another person. Furthermore, the participants perceived that being mum about their unwanted feelings, thoughts, and emotions helped avoid prejudice and judgment. Moreover, they orchestrate their smile to prevent diverting burden to others, maintain their social function, and protect themselves and their reputation.

*“Sometimes, I do not like to disclose my worries because I feel that another person will carry my problem. My problem is my problem, and they have their problem.”* Nursing Student 2.

Also, the participant hides their feeling and emotions due to their social responsibility. In Saudi Arabia, “men hold the most authority and responsibility to ensure the safety and security of their family” [28]. As the participants mentioned, they should not show their weakness because of the expectations of society. Showing their weaknesses makes them feel ashamed and useless since they should be the source of family strength. Thus, the social responsibility of male Saudi nursing student participants is one of the reasons why they need to suppress their emotions.

*“I am ashamed of sharing my problem with my family because I do not want to be seen as a weak person.”* Nursing Student 1.

*“Men are known to be tough, and we should act as the head of the family and protect our family. As young as we are, we are taught to drive because we should be the ones to drive our mother and sister if our father has worked.”* Nursing Student 4.

It was also noted that male Saudi nurses need to protect their family reputations. In Saudi Arabia, “the preservation of honor and community opinion is often at the forefront of Saudi’s minds” [28]. This notion can be associated with the study’s findings that male Saudi nursing students should not show weakness by being emotionally unstable or drained. The participant mentioned that they should not manifest that they have emotional issues and concerns since they will be ostracized or judged in the community. Being considered by other people due to mental health issues is unacceptable and a shame to their families. Thus, they suppress their emotions as a way for them to protect their reputation.

*“I do not share my problem because I am afraid to be judged, and I am worried my masculinity and status will decrease. I am also afraid of what society says, which is a huge problem. If the society thinks I am crazy or delusional, then they will judge me and try to avoid me.”* Nursing Student 7.

Furthermore, the participants mentioned that they do not show their real emotions to preserve their image in society, identity, and dignity. This façade of the participants accords with the cultural expectation from Saudi males that they are tough and in control of themselves.

*“I do not share my problem because another person will use it against me.”* Nursing Student 2.

*“My problem is my problem. I do not have to complain about it, and we do not care about other people’s problems. So, I do not share my problem for my protection.”* Nursing Student 9.

## Discussion

The mental health façade of male Saudi nursing student participants is a reflection of their emotions, feelings, and thoughts. Jung [27] posited that the persons’ attitude or behavior depends on them and their wish to project. As noted, the mental health of the nursing student could be associated with gender norms, culture, individual perspective, and social collectivities. While most participants didn’t talk about hegemonic masculinity, male Saudi nursing students exhibited hegemonic masculinity ideals concerning their mental health. In the context of patriarchy, the participant mentioned that Arabic cultural values, such as men as the head of the family, influenced their mental health. The male guardianship system may have an impact on this perception. The male guardianship system in Saudi Arabia is the authority of a male guardian to make a range of critical decisions for females [29]. Thus, being tough is vital because they are the source of strength for their family and ensure their family’s safety and security [28]. In addition, privacy is a significant concern in Saudi Arabia [30]. Showing or verbalizing emotions among males are signs of weakness in Saudi society. Therefore, the participants must fake their emotions to protect their reputation and self-control. Although this notion is unlikable, suppressing emotions does not evoke others’ unwanted thoughts, feelings, and evaluations [31].

Furthermore, the gender norms in society may serve as challenges to the mental health of the participants. Due to the expected roles of Saudi males in society and their families, they choose to suppress their emotions and exhibit strength despite their internal struggles, which become a barrier for help-seeking behaviors and negatively affect their mental health. Gough and Novikova [32] reported on this phenomenon where the concept of masculinity, as a social construct, often constrains men of their tendency and capacity for emotional communication, help-seeking for mental health, and mental health service engagement. The same study reported that men inclined to traditional masculinity norms are unwilling to disclose their problems to others and ask for help regarding their mental health [32]. Although changing this perception about mental health may be highly challenging due to its deep roots in the society’s cultural, societal, and religious norms and beliefs, the recent societal changes and development that have been implemented in the country are promising. They are seen to be beneficial in advancing and advocating the mental health of Saudi people, especially among younger men. McCrae et al. [33] discussed this observation in a previous review of how the media portray mental health problems in Saudi Arabia. They emphasized the progressive intention of the country to depict mental health problems positively and accurately in society.

The context of hegemonic masculinity could be the possible reason for the participants altering their emotions when dealing with others through suppression or faking of feelings. They suppress their feelings to prevent diverting their burden to others. They believe that sharing their feelings with another person can help them unload their responsibility, but at the same time, it causes a burden to that person. The participants mentioned that unloading a burden on another person is an inappropriate act. Griffiths et al. [34] conducted a qualitative study examining individuals’ perceptions of the advantages and disadvantages of help-seeking from significant others. They reported that help-seeking is viewed to have a negative effect on significant others (i.e., family members or friends) and have an adverse impact on their existing relationships. The participants stressed that they are concerned that seeking help for depression may cause “stress, anxiety, concern, hurt, confusion, and frustration” to their significant others. Similar to the present study’s findings, the participants also expressed their concern that they “burden” their family or friends when they share their problems with them. Griffiths et al. [34] cited another concern: sharing problems with significant others may result in problematic relationships and is described as “placing burden and strain” on such relationships. Nonetheless, the help-seeking and sharing of emotional problems should be encouraged as they are essential methods to cope with mental health problems among young adults [35].

Furthermore, the participants also acknowledged the role of family relationships, which may influence their masculinity. Connell and Messerschmidt [36] mentioned that the pattern of hegemonic masculinity is rooted in families. Saudi male nursing students express the importance of the optimism of their family on mental health that builds their positive attitude toward expressing their behaviors, thoughts, and feelings. Coservano et al. [37] argued that optimism influences positive mental health because people adopt positive behaviors, cognitive responses, problem-solving skills, and understand negative thoughts, feelings, and emotions. Optimism also facilitates the expression of feelings, emotions, and ideas that promote positive mental health.

In this positive sense, optimism can also help in the mutual understanding of people. As mentioned by the participants, mutual guarantee influences their ability to express their emotions. Mutual guarantee is the act of understanding people’s feelings, emotions, and thoughts based on their connectedness and closeness [38]. The present study’s findings reveal that emotions are based on the interaction and developed relationship of a person with another person that makes them understand each other, and their relationship becomes beneficial. This kind of relationship encompasses the promotion of mental health. Weich et al. [39] stated that functional support is significant in mental health promotion.

Moreover, this finding is supportive of “social baseline theory,” which argues “load sharing” as a characteristic of close relationships [40]. That is, emotional burdens are shared across relationship partners. This theory further indicates that human beings have the natural tendency to adapt to be close to other human beings and that people’s functionality decreases when they are apart from their trusted individuals [40]. In applying this theory to the present findings, the participants tend to share their burdens with the people they have quality relationships with; according to the theory, having bad relationships or being distant from significant others results in poor mental health outcomes [40]. This factor was illustrated in the findings of a previous study conducted by Lougheed et al. [41], who suggested that the capacity of individuals to conquer challenging situations is better when they are closer with persons they trust.

## Limitations of the study

The limitations of the study include the focused group of participants. The participants are limited to male nursing students, and other male Saudi non-nursing students might have different stories and social constructs, which may indicate that data saturation is improperly achieved. Also, our study was limited to students who can speak English and are interested in sharing their mental health experiences, leading to possible selection bias toward those who can’t speak English properly. Thus, this study recommends conducting the study in more heterogeneous populations to explore the experiences of mental health.

## Conclusions

The findings document that the mental health façade of male Saudi nursing students is associated with the expectation of family optimism, mutual guarantee, the expectation of society, and self-protection. Participants’ expression of emotions, feelings, and thoughts is based on another person’s behavior, family, and society orientation or awareness on mental health. Moreover, culture and practice influence the mental health façade of the participants.

The findings suggest the need to implement advocacy and community awareness on mental health. The development of community programs, such as seminars in communities about mental health, should be conducted because awareness takes an important role in maintaining the mental health of every individual. Institutions such as schools and universities also conduct male-friendly mental health programs, focusing on promoting mental health for students. The study’s findings may be used to develop these mental health programs for targeting the gender-specific mental health needs of nursing students to ensure that they can enjoy an optimum level of mental health. Moreover, universities should create opportunities to involve male nursing students in discussions about gender norms and how they impact their mental health and well-being to improve their awareness and potentially change their viewpoints. Advocating that seeking help regardless of students’ gender is okay may also assist in improving the emotional communication of male students, thereby improving their help-seeking behaviors. In addition, nursing faculty members should be educated and trained on the gender-specific way of expressing or presenting their mental health problems to recognize and extend help for male students who are reluctant to express their mental health problems because of their fears. Finally, the findings can help the government enhance facilities, policies, and practices, all of which can aid in promoting and awareness of mental health to every citizen.

## Data Availability

The datasets used and/or analysed during the current study available from the corresponding author on reasonable request.
